# Facilitators and Barriers to Breastfeeding and Exclusive Breastfeeding in Kilimanjaro Region, Tanzania: A Qualitative Study

**DOI:** 10.1155/2019/8651010

**Published:** 2019-02-03

**Authors:** Melina Mgongo, Tamara H. Hussein, Babill Stray-Pedersen, Siri Vangen, Sia E. Msuya, Margareta Wandel

**Affiliations:** ^1^Institute of Clinical Medicine, University of Oslo, Norway; ^2^Better Health for African Mother and Child, P.O. Box 8418 Moshi, Tanzania; ^3^Department of Nutrition, Institute of Basic Medical Sciences, University of Oslo, Norway; ^4^Division of Gynaecology and Obstetrics, Oslo University Hospital, Rikshospitalet, Oslo, Norway; ^5^Norwegian National Advisory Unit for Women's Health, Oslo, Norway; ^6^Institute of Public Health, Department of Community Health, Kilimanjaro Christian Medical University College, P.O. Box 2240, Moshi, Tanzania; ^7^Institute of Public Health, Department of Epidemiology and Biostatistics, Kilimanjaro Christian Medical University College, P.O. Box 2240, Moshi, Tanzania

## Abstract

**Background:**

Breastfeeding is the best way to feed infants. It is a simple intervention to improve child health and development. Despite its advantages, there is a low global rate of exclusive breastfeeding (EBF) and, in Kilimanjaro region, Tanzania, EBF is rarely practiced. The aim of this paper is to explore social and cultural factors that might influence the practice of breastfeeding and exclusive breastfeeding in Kilimanjaro region.

**Methods:**

A qualitative design was used. Three districts in Kilimanjaro region, namely, Same, Moshi Municipal Council, and Rombo, were selected. In each district three focus group discussions (FGDs) were conducted with mothers with infants aged 0-12 months.

**Results:**

A total of 78 mothers participated in the focus group discussions. A majority of the mothers were positive towards breastfeeding. They believed that it prevents child sickness, creates happiness, and is good for family economy. Despite the positive attitudes, the mothers revealed many perceptions that interfered with breastfeeding and exclusive breastfeeding. These included the following: breast milk is very light and has bad odor, breastfeeding may affect mothers appearance,* chango* (abdominal pain) has to be treated, there is fear of the evil eye when breastfeeding in public places, breast milk may become unclean, and there is a need of pauses in breastfeeding after the child has burped on the breast.

**Conclusion:**

There are beliefs that promote the practice of breastfeeding in this setting; these local beliefs could be used to develop breastfeeding messages to improve breastfeeding practices. However, there is also a need to address beliefs that interfere with the practice of exclusive breastfeeding in this setting.

## 1. Introduction

Breastfeeding is the best way to provide nourishment to the child [1]. Breastfeeding helps the child to get all the nutrients that are needed for proper growth and development [1–3]. WHO recommends early initiation of breastfeeding, exclusive breastfeeding (EBF), and timely introduction of complementary feeding and continued breastfeeding for up to two years or beyond. EBF means that infants receive only breast milk during the first six months without any additional food or fluid, not even water [1]. The benefits of breastfeeding and EBF for the child, mother, and the community at large are well documented [3–6].

Although breastfeeding is a natural process, it is reported to be influenced by different socio-cultural factors, habits, standards, and behaviors [7–10]. Literature shows that cultural traits related to breastfeeding may be harmful, harmless, or beneficial to the optimal breastfeeding practices [11]. The harmful cultural traits reported to affect optimal breastfeeding practices include: giving prelacteal feeds, discarding colostrum and avoiding breastfeeding after quarreling out of ‘fear of bad blood entering the milk which later may affect the child.' These beliefs and practices are reported to lead to early cessation of EBF and breastfeeding in general [12–14].

In other settings, lack of support from family members or heath care professionals, peer pressure, mothers' body image, the role of women in the reproduction process, and pressure to use artificial feeding have led to early cessation of EBF and breastfeeding [9, 15, 16]. According to Daglas, culture affects the role of women with regard to breastfeeding and may create doubts to the mother's natural ability to feed the baby resulting in early cessation of breastfeeding [15].

In Tanzania, breastfeeding is reported to be universal; about 97% of infants are breastfed and median duration is 21 months. The trend of EBF up to six months at national level has shown increase from 49% in 2010 to 59% in 2015/15 [17, 18]. In the Kilimanjaro region, EBF is reported to be rarely practiced [19]. Different quantitative studies have reported the factors that influence the practice of EBF in the region; the factors include advice on breastfeeding during pregnancy and after delivery, child age, and alcohol intake. However, limited qualitative studies have explored mother's experiences regarding the practice of EBF and cultural traits that may influence EBF. According to Bazzamo and colleagues, culture plays a big role on breastfeeding practices of a given community [20]. In this article culture refers to the context of infant feeding in this community. As the cultural influences may also vary from one setting to another, there was a need to explore the social and cultural influences on the practice of EBF and breastfeeding in the Kilimanjaro region. The findings from this study can give a picture of facilitators and barriers that affect the practice of EBF. The understanding of the facilitators and barriers is important for better planning of interventions that can help mothers adhere to the WHO recommendations for exclusive breastfeeding and breastfeeding practices.

## 2. Methods

This study was conducted by focus group discussions (FGD) with mothers of infants aged 0-12 months in three districts of Kilimanjaro region between August and October 2016. It included a total of 78 mothers in nine focus groups from these districts with three focus groups in each district. The overall project focused on exploring the challenges that women face for practicing EBF. The findings of the first paper focused on women's knowledge, how they related to the advice from health personnel and relatives/friends, and social barriers for EBF [21]. The present paper deals with cultural aspects that have implications for EBF practices.

### 2.1. Study Areas and Recruitment

The three districts were Same, Rombo, and Moshi Municipal Council. Moshi Municipal Council is the capital town for the region and it is more urban compared to Same and Rombo. The major ethnic group in Same is Pare and in Rombo is Chagga. Moshi Municipal Council has mixed tribes.

The field research team comprised five people: two nutritionists, one nurse midwife and two medical doctors. Only four people were involved in running the FGDs [21]. In all three districts we recruited women with the help of village executive officers and community health workers. The inclusion criteria were women who were still breastfeeding and with infants aged 0-12 months. We chose this category of women because they are more likely to be able to recall the practice of exclusive breastfeeding and remember the challenges associated with the practice. We explained the objectives of the study and asked the mothers to give consent to participate in the study. Written consent was obtained from the mothers who agreed and were willing to participate. The focus group discussions were conducted in various settings chosen by the group members, including the ward executive offices, under trees, in a classroom at a school, and in a room at a health centre. Each focus group was comprised of 7-11 mothers and the discussions lasted for one to one and a half hours.

### 2.2. Data Collection

A FGD guide with written questions and probes was used during the discussions. It included questions like the meaning of infant and young child feeding, practices and personal experiences with exclusive breastfeeding, community influences, and practices regarding colostrum, insufficient milk, signs of a hungry child, challenges, and suggestions to improve the practice of exclusive breastfeeding in the community. The focus group discussions were conducted in Swahili and facilitated by a nutritionist who is trained and has experience in FGD. The facilitator is a native Tanzanian and speaks Swahili. All participants were identified by number and not by name, and before the start of each FGD the facilitator discussed with the participants the issue of confidentiality. During discussions, hesitant participants were reassured of anonymity which created a comfortable environment for them to talk. Participants were also informed that the discussions would be tape recorded for later use by the researcher. Further, field notes were taken by research assistants which helped the research team in identifying new information relevant to the study. The field notes were used for triangulation. After each FGD there was a debriefing where the facilitator and the three research assistants discussed the important themes and newly emerging themes. Towards the end of the study there was no new information emerging.

### 2.3. Data Analysis

A thematic analysis was performed according to five steps as described by Braun and Clarke [22]. The first step was familiarization with the data whereby the FGDs were transcribed verbatim and translated to English by the facilitator. All the transcribed FGDs were read by two authors who did the data analysis. During the second step, the authors developed initial codes individually and later compared them with each other's. The developed codes were refined until there was no new code that emerged. The third step was searching for themes. A matrix table was used to list the codes, and all the codes that were related were sorted and listed into one theme. The last two steps involved reviewing and refining the themes and report writing.

### 2.4. Ethics Approval and Consent to Participate

The study was approved by the Kilimanjaro Christian Medical University Ethical Committee (certificate number 916) and Norwegian Regional Ethical Committee (2016/109 REK sør-øst). Permission to conduct research in the respective districts was sought from the District Medical Office and local government authorities. Mothers who participated in the study gave written informed consent.

## 3. Results

In this paper the themes that were mentioned in all nine FGDs are categorized into two sections. Breastfeeding practices included the following subthemes: breastfeeding creates happiness, it is a gift from mother to child, breastfeeding can affect mother's appearance, and breastfeeding is tiring

Facilitators and barriers to EBF practices included the following subthemes: facilitators:* EBF is good for family economy and prevents child sickness, Breastmilk is the only food for infants*

Barriers are* chango* or* makekuu *(abdominal pain that occurs during or after breastfeeding), fear of the evil eye, and burping causing pain to the breast.

### 3.1. Socio-Demographics of Study Participants

The mothers' ages ranged between 19 and 47 with a mean age of 28 years. The majority (65.4%) had primary school education. The participating mothers had a range of 1-7 children, with an average of 3.

### 3.2. Breastfeeding Practices

The mothers in all nine FGDs mentioned that breastfeeding has many benefits. The benefits mentioned included: breastfeeding creates happiness, it is a gift from mother to child, Breastfeeding can affect mother's appearance and breastfeeding is tiring. More details are given below.

*Breastfeeding Creates Happiness*. A majority of the mothers said that the breastfeeding process creates happiness and bonding between mother and child. Breastfeeding makes the mother feel happy. In addition, many mothers said that while breastfeeding they get special attention from other family members and their partners. The mothers-in-law will come to help, and it is at this time that special foods like* mtori *(mashed banana with beef), soups,* and kitawa *(mashed banana with sour milk) are prepared for the breastfeeding mother to enable her to breastfeed properly. Further, the mother gets help with the household chores so that she can relax. When the mother receives this special care, it makes her feel happy and enjoy breastfeeding.

“I enjoy breastfeeding my baby. When my baby is happy I am happy too” FGD 7.

“It's time for me to eat. I get different special foods like soups, porridge so that I can breastfeed …..” FGD 4.

There was a big discussion regarding getting help from other family members. The mothers said that nowadays they can only get help in the first one or two months after giving birth, unlike before when they used to get help so that they could stay at home for six months. The reason is that most of the mothers-in-law are now busy.

“In the past the mother-in-law came to my house to take care of me for six months…nowadays if she comes, it is only for one month, so it's difficult to stay at home and breastfeed” FGD 2.

*A Gift from Mother to Child*. During the discussions some mothers pointed out that breastfeeding is the best gift a mother can give to her baby and it is their role as mothers to breastfeed.

“Since some children refuse to be breastfed, it is a mother's obligation to breastfeed if the child shows that it wants breastmilk” FGD 7.

“It's a way of thanksgiving as other babies refuse to breastfeed”FGD5.

*Breastfeeding Can Affect Mother's Appearance*. The mothers pointed out that breastfeeding could make them gain or lose weight. It was mentioned that some mothers lose weight because they have to breastfeed all the time in order to satisfy the child. And in a society that values women with full-figures, one might not want to lose weight at a certain point. On the other hand, a breastfeeding mother has to eat a lot of food to be able to produce enough milk for her child, which in turn may cause mothers to gain weight and become too fat.

*Breastfeeding Is Tiring*. A few mothers mentioned that breastfeeding was a tiring process. It is associated with fatigue and dizziness which made mothers dislike the whole process of breastfeeding.

“Breastfeeding is tiring and causes dizziness….” FDG 4.

However, some women were of the opinion that certain mothers are simply lazy or have other reasons for choosing not to breastfeed and are making excuses to avoid it.

“Mothers are lazy to breastfeed their children…when the child is breastfeeding and is not satisfied, then the mother says I am tired” FGD 5.

### 3.3. Facilitators and Barriers to EBF Practices

#### 3.3.1. Facilitators to EBF Practice

*EBF Is Good for Family Economy and Prevents Child Sickness*. The mothers pointed out that EBF practice helps to save their money as there is no need to buy formula or cow's milk in the first six months. Furthermore, owing to exclusive breastfeeding, the child does not get sick very often. The participants discussed that mothers with children who are often sick with diseases like coughs, flu, and diarrhea have to visit the hospital frequently. They have to pay for medicine which they sometimes cannot afford; a majority of the mothers have no health insurance. The following citations are from discussions among mothers who had experience from their older children:

“To my experience the child does not cough very often, compared to my previous children when I did not practice exclusive breastfeeding, they used to get flu, coughs… ” FGD 9

“This is my third child…, I followed the advice from the doctor as he told me my child will not suffer from frequent illnesses so I thought that was good and I followed the advice…” FGD 5

“It helps the family economy as the child won't be sick, no need of going to the hospital” FGD 1

*Breastmilk Is the Only Food for Infants*. A few mothers mentioned that breast milk is the only food a child should have in the first six months. They said that they had been informed by healthcare providers during their visits to antenatal clinic that in the first six months the child's stomach is not developed enough to start eating other foods like* mtori,* porridge or* kitawa.*

“My child is three months old and I am still encouraged to practice exclusive breastfeeding. The reason for doing it is that my child's stomach is not yet fully developed and cannot digest other foods”FGD6

#### 3.3.2. Barriers to EBF Practices

In spite of the positive attitudes towards breastfeeding, the FGDs revealed many different issues that were not in favor of EBF. The following issues, elaborated below, were vividly discussed: lightness of breast milk, breastfeeding can affect mother's appearance, breastfeeding is tiring, and breast milk has a bad odor.

*Breast Milk Is Very Light*. All FGD mothers were concerned about the quality of breast milk. They mentioned that breast milk is very light and it cannot fill the child's stomach. This was the reason for early introduction of other foods.

“I cannot practice EBF because the breast milk is very light and the child won't be full. The child feels hunger easily. I have two kids; I had never done exclusive breastfeeding for my kids” FGD 2.

“I understand that the child should be breastfed for six months without mixing but the problem is that the child cries a lot …though the milk is there and the child is breastfeeding, she keeps on crying. I know my milk is very light…and cannot satisfy her. This is the time for having porridge, that's why she is crying. My child is 3.5 months now” FGD 5.

*Breastfeeding Causes Breast Sag*. The young mothers in the groups spoke about how breastfeeding causes their breasts to sag. They were concerned about their appearance; that they would not look good enough for men.

“As a mother I don't find any benefits on practicing exclusive breastfeeding as it causes my breasts to sag” FGD 3.

“In our community young girls like me (aged 18-25 years) do not like to breastfeed. They are afraid that the breasts will sag and their body shapes can change…my friends ask me: why are you breastfeeding? My child is four months now” FGD 8.

*Breast Milk Has Bad Odor*. A few mothers mentioned that breast milk has a bad odor which can also be smelled by others. In addition; the mothers hesitate to express breast milk due to its odor. They also mentioned difficulties with feeding child milk expressed by his/her mother.

“Breast milk has bad odor…It's difficult to give the child the breast milk that has been expressed by his/her mother….” FGD 5.

### 3.4. Circumstances That Make the Breastmilk Unclean

The participants pointed out that the child will get affected if a mother breastfeeds while pregnant. Furthermore, they mentioned that it is prohibited to have extramarital relationships while breastfeeding. They believed that having an extramarital affair or being pregnant makes breast milk unclean and not safe for the child.

“I have practiced EBF to this child. The problem is I cannot have sexual relationship with another man outside my marriage. If it happens that I sleep with another man, my child will be affected. In our language we call it ‘kumkiramtoto' (condition that causes delayed child development and child experiences diarrhea)” FGD 8.

### 3.5. Chango

In all nine FGDs mothers were concerned about a problem with their children which they called* chango. *In other communities they refer to this problem as* makekuu *(means there is dirty blood that causes pain).* Chango* was explained to be the abdominal pain that a child gets during or after breastfeeding. A majority of the mothers said that* chango *occurs when the mother drinks or eats sugary foods like sugarcane, drinks a lot of water during pregnancy, eats cold foods like ice cream or if the child had drunk amniotic fluid while in the womb. The mothers mentioned that* chango* includes some of the following symptoms: the child is born with black lips, has a black line in the middle of the tongue, has a hard tummy, refuses to breastfeed, cries, and has abdominal cramps and stomach rumbling.

“…To me I understand that chango is the dirty stuff that comes from baby stomach when the child is in the womb and it happens that she/he has drunk the water after delivery that will cause chango…” FGD 7.

“…Chango is associated with abdominal pain, excessive baby crying, stomach rumbling; child fails to poop for a day…”FGD 3.

Different methods used to treat* chango, *were mentioned by the mothers. The most common was using traditional herbs. The herbs vary from one community to another, but mostly they include: wild roots of Sodom Apple plant, leaves of certain plants like coffee, and gripe water. The knowledge of traditional herbs has been passed down from one generation to another. Irrespective of the herbs used, the treatment led to early cessation of EBF. Another way to cure* chango or makekuu *was to go to a traditional healer to make abdominal cuttings to the child. The abdominal cuttings are incisions that the child gets around the abdomen for treatment of* chango *or* makekuu.* The incisions are done by an elderly person who has experience. The healer cuts the abdomen until the dark blood comes out, then the healer applies the herbs to the places where there was an incision. If the child does not get healed by this treatment, it will be sent back to the healer, who will then do the incisions on the back.

“My child was crying and my friend told me that my baby had makekuu. I was advised to send the child to the elders and do the abdominal cuttings and the child was also given medicine to lick. Then he got healed” FGD 1.

The belief in* chango *leads to miscommunication between mothers and healthcare providers. The mothers said that health care providers do not believe that there is a health problem called* chango* and this made them seek help from traditional healers for prescription of herbs for their children. In some communities, mothers said that the healthcare providers gave antibiotics to their infants, but that it did not cure* chango*.

“When we go to the hospital we never say the child has chango as the doctors do not understand what chango is. So we just tell the doctor that the child has stomach pain…the doctors give syrups called erythromycin. We buy it in pharmacies, though it does not help. Alternatively we go for traditional remedies” FGD 4.

### 3.6. Fear of Evil Eye

Women fear to breastfeed in public as they could be bewitched by people who have an evil eye. The evil eye is associated with witchcraft. Mothers referred to it as* Zongo.* The evil eye is believed to cause abdominal pain. Therefore, their families have forbidden them to breastfeed in public places.

“We are not allowed to breastfeed outside. People with evil eyes may see the child and she/he may get diarrhea, cry at night and get abdominal distention. In our community we call it ‘zongo'…”FGD 2.

### 3.7. Burping Causes Pain to the Breast

Another issue that could lead to early cessation of EBF was child burping on the mother's breast. The participants maintained that mothers get pain when the child burps on the breast and they cannot breastfeed for a while after that. It was mentioned that a mother is not allowed to breastfeed from the breast that was burped on until she gets treatments. Mothers mentioned that most children burp during the night and it causes pain, cracked nipples or abscesses.

“If the child burps on the breast, the mother is not allowed to breastfeed until the breast is treated. Burping causes swelling of the breast which leads to breast pain” FGD 2.

“The child burped on my breast and I experienced breast pain. To cure this I had to put the breast on my child's head, but it was not cured, I went to the hospital and I found I had a cracked nipple. The nurse at the health clinic advised me to give porridge. My child was only 3months” FGD 2.

## 4. Discussion

This study found that there are many social, beliefs, and traditions that the mothers consider important for their breastfeeding and EBF practices. The themes included the following: breastfeeding creates happiness, it is good for the family economy, prevents child sickness, and breast milk is the only food for the child and a gift that the mother can give to her child. The themes that were not in favor of the practice of EBF were as follows: breast milk is very light, breastfeeding could affect mothers appearance, breastfeeding is tiring, breast milk has a bad odor,* chango*, there is fear of the evil eye, breastmilk may become unclean, and burping causes pain to the breasts.

In this study, mothers mentioned various advantages that relate to the practice of breastfeeding based on their own experiences. Many researchers and WHO have reported similar advantages of breastfeeding [2, 3, 23]. During the first six months, the infant's digestive system is not well matured, and giving other foods may expose infants to infection. Early complementary feeding has been associated with poor nutritional status [24]. From the discussions conducted for this study, it seemed that the women perceived breastfeeding to be the best way to feed infants. The awareness of these benefits can be used to develop educational messages that promote the practice EBF in this setting.

The concern for the quality of breast milk was common in all FGDs. The mothers felt that the milk is very light and cannot satisfy the child. This was the reason for early introduction of complementary foods. A study in Muheza, Tanga, reported that mothers perceived that breast milk alone for the first six months is not enough and that was the reason for early introduction of complementary foods [25]. It has been reported by other researchers that the perception of low breast milk quality made mothers stop breastfeeding [26].

The fear of breasts sagging and body fatness was common among the young mothers. Young mothers feared for the body changes in the whole process of lactation, as it may make them unattractive to men. This problem is alarming especially in developing countries where breastfeeding is important to improve child survival. A study in Kenya showed that young mothers did not want to breastfeed for aesthetic reasons [14]. There is a need for programs that encourage young mothers to breastfeed and to put emphasis on the findings that breastfeeding does not alter breast appearance as documented by other researchers [27, 28].

In this study, mothers reported that breastfeeding is a tiring process and made them lose weight. This negative thought is the reason for early introduction of complementary feeds. Programs that encourage mothers to breastfeed in these communities are needed.

In the present study, mothers reported that breastfeeding while pregnant or while having extramarital affairs was harmful to the baby. The condition was referred to as “*kumkiramtoto*.” The mothers believed that extramarital affairs cause the child to fall sick, mostly with diarrhea and delayed developmental milestones. Studies in Kenya showed that mothers believed that if a mother has extramarital affairs or is pregnant when she breastfeeds, the child will die [14, 16]. In order to prevent the child from dying women who engage in extramarital affairs or are pregnant opt not to continue breastfeeding [14, 16]. Previous study in Tanzania have reported that breastfeeding mothers fear to resume having sex or to have extramarital affairs as this might make the child fall sick. The condition was referred as “*kubemenda*.” The participants believed that having sex while breastfeeding make breast milk unclean [29]. In some cultures mothers were taught to abstain from sex when they are still breastfeeding. Breaking the sexual taboo is a cause of child sickness [30]. The knowledge of such beliefs and attitudes is extremely important in the development of effective breastfeeding educational and promotional messages.

*Chango *was reported to be a major problem during infancy and it interfered with exclusive breastfeeding practices. In some communities it was referred to as* makekuu*. The majority of mothers reported that an infant cries a lot when they have* chango*. In the literature this condition is referred to as colic [31]. The belief that the infant is born with* chango* or* makekuu* made mothers give traditional herbal treatments within the first six months. The traditional remedies given include wild roots of Sodom Apple plant, coffee leaves and abdominal cuttings. The process of abdominal cuttings or incision induces pain to the child. Few mothers opt to give gripe water to calm the baby. There was also miscommunication between healthcare providers and mothers regarding the* chango *problem. The mothers reported that healthcare providers do not believe that* chango *is a problem. Studies in the United Arab Emirates, India, and Nigeria report that mothers gave prelacteal feeds and gripe water to manage infant colic [32]. There is a need for continued health education to mothers on management of colic so that any harmful effects that might be caused by using abdominal cuttings gripe water or traditional herbs can be avoided.

This study collected information about the social and cultural barriers that affect exclusive breastfeeding practices. The study included a variety of women from different ethnic groups and variety of demographics; hence the results of this study can be generalized to these communities. Social desirability bias could be the limitation to the study as some mothers might have withheld what they thought to be negative aspects of their breastfeeding practices. To minimize this, the moderator reminded the mothers about the issues of confidentiality and used probes for mothers to be open regarding the EBF practices. Furthermore, the discussions were moderated by a person who knows the Swahili language and is competent in the field of child feeding. In addition, health care providers working in the health centers were not involved in the FGDs.

## 5. Conclusion

The results of this study revealed that a majority of mothers were positive towards exclusive breastfeeding practices. The positive beliefs towards EBF can be used to develop breastfeeding messages that could encourage mothers to practice EBF.* Chango* or* Makekuu* was seen as a big problem in this community and it interfered with the practice of EBF. The beliefs, knowledge, and remedies to treat* chango* were passed on from one generation to another. There is a need for more research to understand the belief in* chango.*

In this study there were different beliefs and social and cultural barriers that hinder the practice of EBF. The beliefs that hinder the practice are very important to take into consideration when developing interventions to improve breastfeeding practices.

## Data Availability

The data for this study is available from the corresponding author on reasonable request.
